# What are the effects of diagnostic imaging on clinical outcomes in patients with low back pain presenting for chiropractic care: a matched observational study

**DOI:** 10.1186/s12998-021-00403-3

**Published:** 2021-11-23

**Authors:** Hazel J. Jenkins, Alice Kongsted, Simon D. French, Tue Secher Jensen, Klaus Doktor, Jan Hartvigsen, Mark Hancock

**Affiliations:** 1 Faculty of Medicine, Health and Human Sciences, Macquarie University, Rm 347, 17 Wally’s Walk, Sydney, 2109 Australia; 2 Department of Sports Science and Clinical Biomechanics, University of Southern Denmark, Odense, Denmark; 3 Chiropractic Knowledge Hub, Odense, Denmark; 4 Diagnostic Centre – Imaging Section, Silkeborg Regional Hospital, Silkeborg, Denmark

**Keywords:** Diagnostic imaging, Low back pain, Chiropractic, Clinical outcomes

## Abstract

**Background:**

Evidence suggests that diagnostic imaging for low back pain does not improve care in the absence of suspicion of serious pathology. However, the effect of imaging use on clinical outcomes has not been investigated in patients presenting to chiropractors. The aim of this study was to determine if diagnostic imaging affects clinical outcomes in patients with low back pain presenting for chiropractic care.

**Methods:**

A matched observational study using prospective longitudinal observational data with one year follow up was performed in primary care chiropractic clinics in Denmark. Data was collected from November 2016 to December 2019. Participants included low back pain patients presenting for chiropractic care, who were either referred or not referred for diagnostic imaging during their initial visit. Patients were excluded if they were less than 18 years old, had a diagnosis of underlying pathology, or had previous imaging relevant to their current clinical presentation. Coarsened exact matching was used to match participants referred for diagnostic imaging with participants not referred for diagnostic imaging on baseline variables including participant demographics, pain characteristics, and clinical history. Mixed linear and logistic regression models were used to assess the effect of imaging on back pain intensity and disability at two-weeks, three-months, and one-year, and on global perceived effect and satisfaction with care at two-weeks.

**Results:**

2162 patients were included, with 24.1% referred for imaging. Near perfect balance between matched groups was achieved for baseline variables except age and leg pain. Participants referred for imaging had slightly higher back pain intensity at two-weeks (0.4, 95%CI: 0.1, 0.8) and one-year (0.4, 95%CI: 0.0, 0.7), and disability at two-weeks (5.7, 95%CI: 1.4, 10.0), but the changes are unlikely to be clinically meaningful. No difference between groups was found for the other outcome measures. Similar results were found when sensitivity analysis, adjusted for age and leg pain intensity, was performed.

**Conclusions:**

Diagnostic imaging did not result in better clinical outcomes in patients with low back pain presenting for chiropractic care. These results support that current guideline recommendations against routine imaging apply equally to chiropractic practice.

## Background

International back pain guidelines recommend against diagnostic imaging for the management of low back pain in the absence of clinical suspicion of underlying serious pathology (e.g. cancer, infection) or fracture [1–3]. There is no evidence that diagnostic imaging improves clinical outcomes [4–8], although patient satisfaction may be higher if imaging is performed [9, 10]. Conversely, imaging has been associated with poorer patient outcomes, increased downstream healthcare utilisation, and increased healthcare costs [6–8, 11–13]. Despite this, imaging use in the management of low back pain remains common. Recent meta-analyses found that approximately one-quarter of patients with low back pain receive early imaging [14] and approximately one-third of imaging is not in alignment with current guidelines [15].

Studies assessing the impact of diagnostic imaging on patient management and outcomes have typically been performed in general medical care settings and do not show evidence that routine imaging improves clinical management [4]. It may be argued that these results do not extrapolate to patients presenting to chiropractors due to the differing management options being utilised, in particular the common use of spinal manipulative therapy by chiropractors [16, 17] compared to non-physical treatments provided by general medical practitioners [18]. Spinal manipulative therapy uses manual forces through the spine, with possible increased risk for adverse events (e.g. fracture), if there are underlying spinal anomalies or decreased bone density [19]. Therefore, some may argue that imaging may show contraindications to spinal manipulation, thus potentially having more implications for patient management than has been seen with general medical care. However, imaging rarely demonstrates contraindications to spinal manipulation that would be otherwise unsuspected from a thorough clinical assessment, and the usefulness of routine imaging to screen for such contraindications is questionable [20].

Low back pain is the most common reason for patients to present for chiropractic care [17, 21, 22]. Chiropractors commonly use spinal manipulation along with other manual therapies, advice, and exercise prescription to treat people with low back pain [21–23]. X-ray use is common amongst chiropractors, with proportions of use in patients with low back pain ranging from 8 to 84% [20]. Qualitative research demonstrates a belief amongst some chiropractors that imaging can be informative in the management of low back pain by helping to guide optimum delivery or demonstrating contraindications for the use of spinal manipulative therapy [24, 25]. Likewise, there are strong beliefs from some chiropractors that current imaging guidelines are not applicable to chiropractic practice, and in fact, that imaging is required for optimal chiropractic management [25, 26], leading to substantial debate within the profession [27]. To our knowledge, no studies have been performed that assess the effectiveness of imaging on clinical outcomes within chiropractic clinical practice [20]. The aim of this study was to determine if diagnostic imaging had any effect on clinical outcomes (low back pain intensity and disability) in patients with low back pain presenting for chiropractic care.

## Methods

A matched cohort study design was performed using the Danish Chiropractic low back pain cohort (ChiCo) study, a prospective longitudinal observational study with one year follow-up performed between November 2016 and December 2019 [28]. As the ChiCo study followed ethical regulations [28] and only anonymised data was provided for use in this study, the Macquarie University Human Research Ethics Committee provided a waiver to use the ChiCo data without further ethical review.

### Cohort

The ChiCo cohort includes 2818 patients who presented to Danish chiropractors with a new or recurrent episode of low back pain, with or without leg pain, for which they were not in an ongoing course of treatment or long-term management. To be included, patients needed to be 18 years old or over and able to complete electronic questionnaires in Danish. Patients with a confirmed diagnosis of fracture, infection, cancer or other serious pathology were excluded from the ChiCo study. Baseline questionnaires were completed by both participants and chiropractors, and participants underwent a baseline clinical assessment with the treating chiropractor. Follow-up questionnaires were completed by participants at two weeks, three months, and one year. Treatment was provided as needed and not impacted by participation in the study. No limitation was placed on access to other healthcare services. For the current study, participants identified by the chiropractor at baseline to have previous imaging relevant to their current clinical presentation were excluded (N = 656). All other participants in the ChiCo study were eligible for inclusion (N = 2162).

### Data collection

Baseline assessment for each participant included two patient questionnaires and a clinical assessment questionnaire completed by the treating chiropractor. The first patient baseline questionnaire was performed at the clinic prior to assessment with the chiropractor. This baseline questionnaire included: age; sex; low back and leg pain intensity, measured on a 0–10 numerical rating scale; low back disability, measured using the Roland Morris Disability Questionnaire (RMDQ), with results proportionally recalculated to a score from 0 to 100; duration of current episode of low back pain (1–2 days; 3–7 days; 1–2 weeks; 2–4 weeks; 1–3 months; 3–12 months; more than 1 year); risk of poor prognosis, measured using the STarT back tool; previous imaging for back pain (yes/no); and expectations about the use of imaging within the initial assessment (yes/no). The second patient baseline questionnaire was performed immediately after the initial assessment, and included: previous treatment for back pain (surgical or non-surgical); and existing co-morbidities. The clinical assessment questionnaire completed by the chiropractor at the first visit included: suspicion of serious pathology as the cause of low back pain (yes/no); low back pain diagnosis (non-specific low back pain, spine-related leg pain with nerve root involvement, spine-related leg pain without nerve root involvement, or suspected fracture, infection, cancer, cauda equina syndrome, or inflammatory arthritis); pre-existing and relevant imaging for the presenting low back pain (yes/no); intention to treat the patient with high velocity, low amplitude manipulation (yes/no); and whether they referred the participant for imaging (X-ray, CT, or MRI).

Follow-up questionnaires were completed online via REDCap by participants at two weeks, three months, and one year. All follow-up questionnaires included the low back pain numerical rating scale and RMDQ. In addition, the two-week questionnaire included items on satisfaction with care and global perceived effect on low back pain.

### Diagnostic imaging

Participants who were referred for diagnostic imaging (X-ray, CT, or MRI) by the chiropractor at the first visit were defined as the exposure group. Participants who were not referred for imaging at the first visit were defined as the non-exposure group.

### Matching variables

Matching was performed using baseline variables which were considered by the research team to potentially affect the decision to refer for imaging and were also associated with outcome measures on regression analysis. Baseline variable selection was informed by the literature on reasons chiropractors refer for imaging [20, 25, 29–31], and the expert opinion and clinical experience of the research team. The baseline variables that were assessed for association with outcome measures were: age; sex; low back pain intensity; duration of low back pain; leg pain intensity; low back disability; risk of poor prognosis (STarT back tool); previous imaging; previous surgery; previous low back pain treatment (surgical or non-surgical); chiropractor suspicion of serious pathology; chiropractor intention to treat with high velocity low amplitude spinal manipulation; participant expectations of imaging; and the presence of co-morbidities.

### Outcome measures

The primary outcomes were participant reported low back pain intensity and disability at three months.

Secondary outcomes were participant reported low back pain intensity and disability at two weeks and one year, and global perceived effect on low back pain and satisfaction with care at two weeks. Global perceived effect was measured on a seven-point Likert scale from ‘much better’ (response 1) to ‘much worse’ (response 7). Responses were dichotomised for analysis to ‘improved’ (responses 1 or 2, ‘much better’ or ‘better’) or ‘not improved’ (responses 3–7, ‘slightly better’ to ‘much worse’). Satisfaction with care was measured on a five-point Likert scale from ‘to a very high degree’ (response 1) to ‘not at all’ (response 5). Responses were dichotomised for analysis to ‘satisfied’ (responses 1 or 2, ‘to a very high degree’ or ‘to a high degree’) or ‘not satisfied’ (responses 3–5. ‘to some degree’ to ‘not at all’).

### Missing data

Missing baseline data were imputed using multiple imputation in SPSS (IBM Corp. Released 2017. IBM SPSS Statistics for Windows, Version 25.0. Armonk, NY: IBM Corp.), depending on the type and reason for the missing data. Twenty-seven percent of ChiCo participants did not receive the second baseline questionnaire to complete. Therefore, data related to previous treatment and co-morbidities were imputed for these participants. Missing data were also imputed for baseline leg pain intensity (2.3% missing), low back pain intensity (2.0% missing), and duration of back pain (0.7% missing). Participants with missing outcome data were removed from the analyses.

### Data analysis

To account for potential differences between the groups that may have impacted on outcomes, the exposure and non-exposure groups were matched on baseline variables using coarsened exact matching (CEM) [32, 33]. In CEM, exact matches of participants in the exposure (imaging) and non-exposure (no imaging) groups were compared. However, as it was not realistic to match exactly on continuous variables, the data were ‘coarsened’ to allow exact matching in pre-determined categories. Cut-points to define the strata for matching were selected for age (18–50, 51–70, greater than 70 years), low back pain intensity (0–3, 4–7, 8–10 on the numerical rating scale); low back disability (0–20, 21–40, 41–60, 61–100 on the RMDQ); and leg pain intensity (0–3, 4–7, 8–10 on the numerical rating scale). CEM assigns individuals to strata of baseline characteristics and discards any strata that do not include at least one exposed and one non-exposed participant. Each stratum may contain different numbers of exposed and non-exposed participants. This matching was used in the subsequent effect estimation while applying weights to compensate for differences in strata size.

Baseline variables in the exposure and non-exposure groups were presented descriptively. Observed and CEM-weighted standardised differences in means between the exposed and non-exposed groups were calculated for baseline variables used in the matching process.

The effect of imaging was estimated using mixed models with random effects of chiropractors and CEM-weightings. The primary model was adjusted for the outcome measure at baseline (low back pain intensity or disability) as well as for duration of pain. Sensitivity analyses were performed with additional adjustment for covariates that did not achieve almost perfect balance with CEM matching (standardised difference in means of zero). Mixed linear models were used for continuous outcomes (back pain intensity and disability), and mixed effect logistic regression was performed for dichotomous outcomes (global perceived effect and satisfaction with care). Separate models were conducted for each time point (two weeks, three months, and one year). All data analyses were performed using STATA (StataCorp. 2019. Stata Statistical Software: Release 16. College Station, TX: StataCorp LLC).

## Results

Of the 2,162 participants included in this study, 874 (40.4%) were female, the mean age was 43.3 years (SD: 13.4), and 522 (24.1%) were referred for imaging at the first visit. Of those referred for imaging, 505 (96.7%) were referred for X-ray alone, 8 (1.5%) for X-ray and MRI, 7 (1.3%) for MRI alone, and 2 (0.4%) for X-ray, CT, and MRI. The baseline characteristics of participants referred and not referred for imaging are presented in Table 1. Participants referred for imaging were more likely to be female (43.3% vs. 39.5%), older (mean age: 49.4, SD 14.6 vs. 41.4, SD 12.4), have more than 3 months pain duration (30.3% vs. 9.7%), expect an imaging referral (39.7% vs. 20.8%), have high risk of a poor prognosis (27.8% vs. 18.0%), report at least one comorbidity (45.4% vs. 35.3%), and have clinical suspicion of underlying serious pathology (4.6% vs. 0.4%). The CEM-weighted standardised difference in means approached zero across the covariates used for matching; however, perfect balance was not obtained for age and leg pain with standardised difference in means of 0.1 (Table 1). Expectations of imaging and the presence of co-morbidities were assessed as potential baseline matching variables; however, regression analyses found no consistent association between these variables and the outcome measures, so they were removed from the analysis.

**Table 1:** Baseline variables stratified for those receiving or not receiving an imaging referral

Baseline variable	Imaging referral N = 522	No imaging referral N = 1640	Standardised difference in means	
			Observed^#^	CEM-weighted^†^
Female* (N, %)	226 (43.3)	648 (39.5)	0.1	0.0
Age* (mean, SD)	49.4 (14.6)	41.4 (12.4)	0.6	0.1
Back pain intensity* (mean, SD)	6.7 (2.0)	6.7 (2.1)	0.0	0.0
Leg pain intensity* (mean, SD)	3.7 (3.2)	2.5 (2.7)	0.4	0.1
Disability* (mean, SD)	55.1 (23.2)	54.2 (24.3)	0.0	0.0
Pain duration*			0.6	0.0
Less than 2 weeks (N, %)	220 (42.1)	1131 (69.0)		
2 weeks to 3 months (N, %)	144 (27.6)	350 (21.3)		
Over 3 months (N, %)	158 (30.3)	159 (9.7)		
Expectation of imaging referral (N, %)	207 (39.7)	341 (20.8)		
STarT back risk*			0.3	0.0
Low (N, %)	191 (36.6)	759 (46.3)		
Medium (N, %)	186 (35.6)	586 (35.7)		
High (N, %)	145 (27.8)	295 (18.0)		
Previous imaging* (N, %)	143 (27.4)	434 (26.5)	0.0	0.0
Previous surgery* (N, %)	16 (3.1)	35 (2.1)	0.1	0.0
Previous treatment* (N, %)	330 (63.2)	1013 (61.8)	0.0	0.0
Presence of any comorbidity (N, %)	237 (45.4)	579 (35.3)		
Presence of lumbar spine comorbidity (N, %)	94 (18.0)	146 (9.5)		
Suspicion of serious pathology* (N, %)	24 (4.6)	7 (0.4)	0.4	0.0
Intention to use manipulation* (N, %)	488 (93.5)	1596 (97.3)	− 0.2	0.0

^*^Baseline variables used in CEM matching

^#^Standardised difference in means of the whole (unmatched) population

^†^Standardised difference in means of the CEM-weighted (matched) population

Table 2 presents the overall observed outcomes, stratified for those that either received or did not receive an imaging referral. Low back disability was higher in those that received an imaging referral at all time points. Global perceived effect of ‘much better’ was lower in those that received an imaging referral at 2-weeks follow-up. All other outcomes were similar between those that received or did not receive an imaging referral.

**Table 2:** Observed outcomes stratified for those receiving or not receiving an imaging referral

Outcome measure	Imaging referral	No imaging referral
*Primary outcomes:*		
Back pain 3 months; N = 1437 (Mean, SD)	2.6 (2.3)	2.0 (2.2)
RMDQ 3 months; N = 1286 (Mean, SD)	23.1 (23.0)	15.5 (21.7)
*Secondary outcomes*		
Back pain 2 weeks; N = 1501 (Mean, SD)	4.2 (2.3)	3.4 (2.2)
RMDQ 2 weeks; N = 1511 (Mean, SD)	35.9 (26.3)	25.2 (24.2)
Back pain 1 year; N = 1368 (Mean, SD)	2.7 (2.6)	2.0 (2.3)
RMDQ 1 year; N = 1114 (Mean, SD)	22.3 (22.5)	16.6 (21.4)
Global perceived effect (N)	375	1093
Much better (N, %)	110 (29.3%)	475 (43.5%)
Better (N, %)	123 (32.8%)	355 (32.5%)
A little better (N, %)	73 (19.5%)	151 (13.8%)
Almost the same (N, %)	55 (14.7%)	85 (7.8%)
A little worse (N, %)	6 (1.6%)	13 (1.2%)
Worse (N, %)	4 (1.1%)	11 (1.0%)
Much worse (N, %)	4 (1.1%)	3 (0.3%)
Satisfaction with care (N)	382	1099
To a great extent (N, %)	107 (28.0%)	357 (32.5%)
Greatly (N, %)	182 (47.6%)	525 (47.8%)
Somewhat (N, %)	66 (17.3%)	173 (15.7%)
To a small extent (N, %)	16 (4.2%)	28 (2.5%)
Not at all (N, %)	4 (1.0%)	9 (0.8%)
Do not know (N, %)	7 (1.8%)	7 (0.6%)

### The effect of imaging using CEM-weightings

The results from the mixed models with CEM weightings are presented in Table 3. For the primary analysis 720 participants were matched for low back pain intensity at three months (185 in the exposure group and 535 in the non-exposure group) and 731 participants were matched for disability at three months (189 in the exposure group and 542 in the non-exposure group), with minor and statistically non-significant differences in outcomes between groups. The imaging exposure group had slightly higher low back pain intensity at two weeks (0.4; 95% CI: 0.1, 0.8; 198 in exposure group, 553 in non-exposure group) and one year (0.4; 95% CI: 0.0, 0.7; 152 in exposure group; 415 in non-exposure group), and slightly higher low back disability at two weeks (5.7; 95% CI: 1.4, 10.0; 224 in exposure group, 627 in non-exposure group). For all other outcomes, differences were minor and statistically non-significant. Similar results were found with sensitivity analysis.

**Table 3:** CEM-weighted outcomes in exposure (imaging) and non-exposure (no imaging) groups: including matched participants only

Outcome measure	Matched sample size	Unadjusted model	Adjusted model	Sensitivity analysis^^^
	Imaged (N)/Not imaged (N)	Effect size (95% CI)	Effect size (95% CI)	Effect size (95% CI)
Primary outcomes:				
Low back pain intensity 3 months*	185/535	0.2 (− 0.2, 0.5)	0.1 (− 0.2, 0.4)	0.1 (− 0.2, 0.5)
Disability 3 months^#^	189/542	0.2 (− 4.4, 4.8)	0.8 (− 3.2, 4.8)	0.8 (− 3.1, 4.7)
Secondary outcomes:				
Low back pain intensity 2 weeks*	198/553	0.5 (0.2, 0.8)	0.4 (0.1, 0.8)	0.5 (0.1, 0.8)
Disability 2 weeks^#^	224/627	4.2 (− 1.1, 9.5)	5.7 (1.4, 10.0)	5.9 (1.4, 10.4)
Low back pain intensity 1 year*	152/415	0.5 (0.1, 0.9)	0.4 (0.0, 0.7)	0.4 (0.1, 0.7)
Disability 1 year^#^	160/468	− 2.9 (− 6.8, 1.0)	− 2.1 (− 5.7, 1.5)	− 2.4 (− 6.0, 1.2)
Global perceived effect 2 weeks^†^	150/369	0.8 (0.5, 1.4)	0.8 (0.5, 1.3)	0.8 (0.5, 1.3)
Satisfaction with care 2 weeks^†^	150/369	0.9 (0.5, 1.6)	0.9 (0.5, 1.7)	0.9 (0.5, 1.7)

^*^Low back pain intensity numerical rating scale measured out of 10. A positive coefficient indicates higher pain in the exposure group. Adjusted for baseline low back pain and duration of pain

^#^Low back disability RMDQ measured out of 100. A positive coefficient indicates higher disability in the exposure group. Adjusted for baseline low back disability and duration of pain

^†^Global perceived effect and satisfaction with care dichotomised. An odds ratio greater than one indicates higher improvement or satisfaction in the exposure group. Adjusted for baseline low back pain, disability, and duration of pain

^^^Sensitivity analysis additionally adjusted for covariates that did not obtain perfect balance: age, leg pain intensity

## Discussion

To our knowledge, this is the first study that has assessed the effect of diagnostic imaging for low back pain patients who present to chiropractic care. This study found that patients with low back pain presenting for chiropractic care who received diagnostic imaging had similar clinical outcomes when compared to matched patients who did not receive diagnostic imaging. For secondary outcomes, there was a trend towards poorer outcomes among the patients who received imaging; however, differences were small and unlikely to be clinically important.

Strengths of this study include the use of prospective longitudinal observational data from a large sample of care-seeking patients with back pain. Statistical methods were used in this study to use observational data to investigate causal effects by matching exposed and unexposed participants on multiple observed baseline variables [34], thus approximating the effect of randomisation. The baseline variables selected for matching were predetermined as variables likely to impact clinical decision-making to refer for imaging and related to the outcomes investigated. To account for any baseline variables that did not achieve almost perfect balance in the matching process sensitivity analyses were performed, adjusting for these variables. The estimated effect on outcomes found in this study relates to patient profiles where there was variation in clinician decision-making to use or not use imaging. Therefore, this study estimates the effect of imaging resulting from clinician decision-making, rather than the effect of being randomised to imaging, regardless of clinician preference as may occur in a randomised controlled trial.

A limitation of this study is that unmeasured variables cannot be accounted for. Randomisation ideally results in groups that are similar for both known and unknown variables [34], whereas observational data can only be matched on known, measured variables. When matching is performed, participants that cannot be matched on the measured variables are removed from analysis, decreasing sample size. Although this allows the comparison of groups that could have received imaging, it also decreases the certainty of results and increases confidence intervals. In this study, sample size in the exposure group for the primary outcomes was 185 for pain intensity and 189 for disability (with 535 and 542 in the non-exposure group respectively). Post hoc analysis of the primary analysis found these sample sizes sufficient to detect confidence intervals with widths of 0.7 points (out of 10) for low back pain intensity and 9.2 points (out of 100) for disability. These differences are generally considered to be below a smallest worthwhile effect. Therefore, sample size was determined sufficient to assess the aims of this study. Data imputation was used for some baseline variables where there was less than 5% missing data, and for the 27% of participants who did not receive the second baseline survey. Imputation was considered suitable for the second baseline survey despite the high percentage of missing responses as the missing data was not related to the participant or the participants choice to complete the questionnaire. No imputation was performed on outcome variables, and participants with missing outcome variables were excluded from the analysis. Referral for all types of diagnostic imaging was assessed; however, chiropractors only referred for CT or MRI in 3.3% of cases, meaning that the results predominantly reflect the use of X-rays. While this limits conclusions that can be drawn regarding the effectiveness of using CT or MRI specifically, it is reflective of imaging use in chiropractic clinical practice where X-rays are commonly used [20]. Finally, it is possible that participants in the non-exposure group may have later received an imaging referral during the follow-up period. The effect this may have had on outcomes cannot be specifically accounted for; however, results were consistent across the follow-up time points, suggesting that there was minimal effect.

The results of this study suggest that the effectiveness of imaging in patients who seek care from chiropractors for low back pain is similar to results observed in those who seek medical care. Meta-analyses of six randomised controlled trials assessing the effectiveness of imaging for low back pain in patients seeking care from medical doctors [4] found a small, but clinically unimportant difference in pain, in both the short and long term, in favour of the no-imaging group. Some observational studies have been performed assessing the effect of early imaging on clinical outcomes using propensity-score matching [8] or CEM [7], or assessing associations between early imaging and clinical outcomes using multivariable regression analysis [6]. These studies were performed using large claims databases in specific populations, including older adults [8], veteran affairs [7], and workers compensation [6]. Similar to the current study, these previous studies found either small and clinically unimportant differences in pain or disability in favour of the no-imaging group [6, 7], or no differences between groups [8].

Previous randomised controlled trials have found that patients receiving diagnostic imaging have a higher satisfaction with care [9, 10]. In the current study no difference between groups was found for either global perceived effect or satisfaction in care. Clinicians have reported that they may be more likely to refer for imaging to help reassure the patient or increase their satisfaction with care [35], conflicting with the results of our study. Explanations for the different results in the current study could include the lower initial expectations for imaging in participants who did not receive imaging, or chiropractors may have been able to adequately reassure participants using other methods when imaging referral was not indicated.

The results of this study support that recommendations from current clinical guidelines for the use of imaging for low back pain are equally relevant for patients presenting for chiropractic care as for those patients presenting to other health professionals [1–3]. The chiropractors in this study planned to use spinal manipulative therapy with the majority of participants (96.4%), suggesting that, contrary to the beliefs of some chiropractors, imaging does not appear to improve clinical outcomes in patients receiving spinal manipulative therapy. Imaging may be more frequently requested by some chiropractors who perceive it can reduce possible adverse events and assist in providing spinal manipulative therapy safely [19, 20]. Significant adverse events from lumbar spinal manipulative therapy are rare [36], and a much larger participant cohort than that included in the the ChiCo study would be required to accurately measure incidence and baseline predictors of significant adverse events. Therefore, a measure of adverse events was not obtained in the ChiCo study and the effect of obtaining imaging on adverse events cannot be directly assessed. However, if adverse events were more common in the group that did not receive imaging, it would seem likely that there would be lower global perceived effect and/or satisfaction with care in that group, which was not observed in the current study.

## Conclusions

In this observational study, diagnostic imaging did not result in better clinical outcomes in patients with low back pain presenting for chiropractic care. Conversely, there was a trend towards slightly poorer outcomes in the imaging group; however, differences were of unlikely clinical importance. These results suggest that current guideline recommendations against routine imaging apply equally to chiropractic practice.

## Data Availability

ChiCo data are stored and managed at Chiropractic Knowledge Hub (formerly the Nordic Institute of Chiropractic and Clinical Biomechanics) with the University of Southern Denmark (SDU) as the responsible data authority (Danish Data Protection Agency, j.nr.: 2015–57-0008/16–47,215). The datasets used and/or analysed during the current study are available from the corresponding author on reasonable request.

## Abbreviation

CEM: Coarsened exact matching
